# Perceived Role, Identity and Experiences of Pharmacists and the Potential Impact on COVID-19 Vaccine Uptake per Louisiana Region: A Prospective Cohort Study

**DOI:** 10.3390/ijerph20156459

**Published:** 2023-07-27

**Authors:** Brittany A. Singleton, Sara Al-Dahir, Christopher Gillard, Martha Earls, Julia Bommarito, Malcolm Duhe, Kevin Phi

**Affiliations:** Division of Clinical and Administrative Sciences, Xavier University of Louisiana, New Orleans, LA 70125, USA

**Keywords:** COVID-19, pharmacy-based vaccination services, pharmacist perspectives, frontline healthcare workers

## Abstract

Some of the lowest COVID-19 community vaccination rates in America are found in Louisiana. This study investigated: (1) barriers that Louisiana pharmacists encountered during the pandemic; and (2) the effect of pharmacists’ role and identity confidence on willingness to enforce vaccine mandates, and COVID-19 vaccine uptake. Fifty-four community pharmacists from nine regions of Louisiana participated in the study. Pharmacists completed questionnaires about: personal demographics, patient population, vaccination encouragement, COVID-19 concerns, and vaccination administration rates. The importance of feeling like a trusted voice in the community, as well as professional perception and self-assurance, were measured using Likert scale questions. During focus groups, participants discussed experiences with the COVID-19 vaccination rollout and vaccination-related obstacles. As the pandemic progressed, pharmacists reported being overworked, understaffed, and overburdened with new responsibilities. In regions with lower vaccination rates, pharmacists were less likely to feel at ease enforcing vaccine mandates. Independent pharmacists were less comfortable enforcing vaccine mandates than chain pharmacists but had more positive perceptions of their role and identity. This study contributes to further understanding of pharmacy workflow obstacles and pharmacists’ perceptions of their professional roles and identities in the community.

## 1. Introduction

For the past three decades, United States pharmacies have been widely used vaccine administration settings, and American pharmacist duties continue to expand [1]. From common vaccines such as the annual flu vaccine, to more population specific vaccines, such as the pneumonia or shingles vaccines, an increasing number of patients are receiving their vaccines from the same provider that fills their prescriptions [1]. The American Pharmacists Association’s 2019 Guidelines for Pharmacy-based Immunization strongly encourages pharmacists to be vaccine educators and advocates [2]. Prior studies regarding the impact of pharmacy-based vaccination services have shown that pharmacist’s interventions as vaccine educators, facilitators, and immunizers are effective at increasing community vaccination rates [3,4]. From a public health perspective, community pharmacies can play a vital role in that pharmacists can act as immunizers, improve vaccine-related health literacy, and increase vaccination coverage rates [5]. However, multifactorial challenges to pharmacy-based vaccination services remain.

### 1.1. Workload and Compensation

American Community pharmacists’ roles as immunizers continues to expand as the workload of retail pharmacies skyrockets. There is an ever-increasing prescription fill load which was on the rise before the COVID-19 pandemic [6]. Pharmacists’ prescription fill workloads are increasing, in part, because pharmacy administrators may place a strong emphasis on prescription volume among other profit generating functions [7]. This ultimately leaves little time for other essential pharmacy tasks such as patient counseling, employee training, immunization administration, medication therapy management, and communication with providers [8,9]. Higher volumes of prescriptions have been linked to higher levels of pharmacist burnout [6]. Current pharmacy working conditions have been cited as a major reason for pharmacist burnout and a recent shortage of retail pharmacists [10]. The COVID-19 pandemic has exposed these issues drastically, where a massive rollout of new vaccines combined with existing workload demands placed a significant amount of strain on pharmacy staff.

Pharmacies have been on the frontline of the COVID-19 vaccine rollout and administration. According to information provided by the U.S. Centers for Disease Control and Prevention (CDC), over 70% of COVID-19 vaccinations have occurred in pharmacies [11]. Certified immunizer pharmacists and pharmacy technicians were tasked with the prescription fill workload while also attempting to keep up with vaccine demand [12,13]. The administration of COVID-19 vaccines also occurred alongside influenza vaccinations during the fall and winter months, adding another layer of work to the already overworked pharmacy staff [14]. Within the strained and often understaffed pharmacy settings, financial incentives were not offered to workers. Overall lack of compensation for increased workload and low insurance reimbursement rates posed significant issues for immunizing pharmacies [15,16,17].

### 1.2. Accessibility

Accessibility has been a challenge to pharmacy-based vaccination services. For nearly two decades, drug supply shortages have challenged pharmacy staff at an increasing rate [18,19]. Such shortages can adversely affect drug therapy and medical procedures, potentially resulting in medication errors [20]. At the start of the COVID-19 pandemic, existing drug supply chain issues multiplied. Pharmacist survey data from the National Community Pharmacists Association indicate that almost 90% of respondents were affected by supply chain issues [21]. During the COVID-19 vaccine rollout, many pharmacies experienced difficulties obtaining the vaccine. Lack of accessibility, especially in rural areas of the U.S., was a major obstacle to vaccinating the public. Some pharmacies experienced frequent shipping delays, which may have resulted in missed opportunities to provide care [22]. There was a disproportionate impact in rural areas, where independent pharmacies may be the only vaccination provider [23]. Natural disasters have also impacted COVID-19 vaccination access, particularly in the state of Louisiana. In 2021, Hurricane Ida made landfall in Louisiana amid a COVID-19 surge [24]. Subsequent road closures, structural damage, loss of staffing, and lack of electricity forced some pharmacies to close [25,26]. Therefore, prescription fills and vaccinations were delayed or halted altogether.

### 1.3. Patient-Specific Factors

Vaccine hesitancy, which refers to a delay in acceptance or refusal of vaccination despite availability of vaccination services, plays a major role in low vaccination rates seen across the United States in both primary care settings and pharmacies [27]. Significant vaccine hesitancy exists regarding the COVID-19 vaccines [28]. Because many vaccines take years to research and develop, patients were concerned about the perceived rapid development of the COVID-19 vaccine [29]. A lack of knowledge and understanding regarding mRNA technology fueled further COVID-19 vaccine concerns [29]. Many patients within the United States and abroad express concerns regarding the potential side effects of the COVID-19 vaccines [30,31]. Due to the politicization of the COVID-19 pandemic and vaccines, many hesitant patients had a growing sense of mistrust in the government-led vaccination campaign [28,29]. There is also misinformation surrounding COVID-19 and the associated vaccines, which were widely spread on the internet and in the media [32,33]. These patient-specific factors, in combination with preexisting healthcare system mistrust, have ultimately led to lower vaccination rates.

### 1.4. Louisiana: A COVID-19 Vaccine Hesitancy Stronghold

Louisiana pharmacists serve in some of the most vaccine resistant regions of the United States, with Louisiana ranking poorly when considering overall vaccination rates [34]. There are approximately 6000 practicing pharmacists within the state of Louisiana [35]. The state of Louisiana is home to 64 parishes (counties) separated by the Louisiana Department of Health (LDH) into nine different administrative regions [36]. While each is designated by geography and population, certain regions or groups of regions are similar based on demographic composition, culture, and historical identity [37]. Healthcare landscapes, associated behaviors, and health outcomes also vary from region to region [38]. Louisiana currently has one of the lowest percentages of fully vaccinated people nationally; of the 50 states, Louisiana is currently ranked as number 46 of 50 [34]. The first COVID-19 vaccine was given Emergency Use Authorization (EUA) by the U.S. Food and Drug Administration (FDA) in December 2020, and the first COVID-19 vaccine was fully FDA-approved in August 2021 [39]. In the year since the Pfizer-BioNTech COVID-19 vaccine was given full FDA approval, Louisiana’s vaccination rate increased by only 10%, from 34% to 44% [40]. This minimal increase in COVID-19 vaccinations occurred despite available incentives and workplace-imposed mandates.

Pharmacists have consistently been rated by Americans as one of the most trusted healthcare professionals for their honesty, ethical standards, and accessibility in the community [41]. Being a trusted voice to patients is crucial, especially in community pharmacy settings. It is also crucial to consider pharmacist perspectives regarding their role within the community, their self-perceived professional identity, and their practice-based experiences, particularly regarding pharmacy-based immunization services. Using a mixed methods approach, this study explored Louisiana community pharmacists’ confidence in their professional roles and identities, as well as their comfort in enforcing vaccine mandates, and how these perceptions may have impacted COVID-19 vaccine uptake in their region. This study also explored the specific barriers that Louisiana pharmacists have faced during the COVID-19 pandemic and its impact on COVID-19 vaccination rates by region.

## 2. Methods

### 2.1. Pharmacist Recruitment

This was a prospective cohort study among community pharmacists within the state of Louisiana. Community pharmacists who provide COVID-19 vaccinations in their daily practice were recruited. A convenience sample was collected from this cohort in 2021. An announcement was sent to Louisiana pharmacists affiliated with Xavier University of Louisiana who provide experiential education to pharmacy students. In addition, pharmacies in various areas of the state were sent the announcement and targeted for recruitment on the basis of Louisiana Department of Health designated health regions. There was an effort to target community pharmacies in Louisiana regions with lower overall COVID-19 vaccination rates. All pharmacists were vaccinators and served in pharmacies that provided COVID-19 vaccines and other vaccines as part of routine patient care.

### 2.2. Surveys

Each pharmacist completed a baseline survey, participated in a focus group discussion, completed an online COVID-19 vaccination education course, and completed an abbreviated follow up survey. Electronic surveys were conducted from May 2021–May 2022. Survey domains included demographics, professional education and training, immunization and practice setting, employer vaccine mandates, and personal impact of the COVID-19 pandemic. Additional domains included professional role and identity as a trusted voice in their respective communities, perceived moral responsibilities and obligations in serving patients, vaccination attitudes, COVID-19 vaccine concerns, and comfort in vaccinating patients relative to vaccine mandates. The researchers developed original, closed ended questions for the surveys. A reliability analysis was conducted around each scale and each consistently resulted in a Cronbach Alpha score >0.7. A Role and Identity variable was measured through Likert scale questions (5 being the most positive and 1 being the most negative) regarding how the pharmacists perceived the importance of their position, if they felt like trusted voices in the community, and their professional perceptions and confidence. Comfort was defined as the professional willingness to recommend or administer a vaccine. Comfort administering a COVID-19 vaccine relative to a mandate was also measured through Likert scale questions (5 being the most comfortable and 1 being the least comfortable). Each domain had a minimum of 5 questions.

### 2.3. Focus Groups

Each pharmacist also participated in a 90-min virtual focus group centered on COVID-19 experiences and COVID-19 vaccination-related barriers. Additional questions focused on workflow issues, commonly encountered vaccine concerns from the community, and potential solutions to increase vaccine uptake in the pharmacy setting. Twelve total focus groups were conducted from September–October 2021, and again in October 2022. A combination of conversation transcription and polling technology was used to collect data from the focus groups. Focus groups were electronically recorded, automatically transcribed, and manually reviewed for accuracy of transcription. All pharmacists were compensated for both their survey completion and focus group participation in the form of a gift card.

### 2.4. Louisiana Regions

Pharmacists were recruited from each of the nine Louisiana Department of Health (LDH) administrative regions. COVID-19 vaccine uptake rates were captured for each region using data from the LDH. These geographical regions were stratified into groups of three (Tertiles) based upon their inhabitants’ COVID-19 vaccine uptake, with those three administrative regions falling within the lowest vaccination percentage rate categories being placed into Tertile 1 (T1), those three falling into the intermediate range being placed in Tertile 2 (T2), and those three regions with the highest vaccination rates being placed into Tertile 3 (T3). Vaccination rates per Tertile were measured during the month of May in years 2021 and 2022. These vaccination rate values were compared to pharmacist survey response data, as well as measured against each other to assess relative change in vaccination rates across regions.

### 2.5. Data Analysis

All baseline variables are expressed as nominal or continuous variables. Nominal variables are provided as number and percent. Continuous variables are provided as mean and standard deviation. Each domain for pharmacist-perceived confidence and professional identity were converted to summative scores and normalized to the 5-point scale, with 1 representing the lowest self-perception, i.e., most negative response and 5 representing the most positive response. A reliability analysis was run across the major professional and perception domains, with Cronbach alpha’s ranging from 0.81 to 0.85, indicating a reliable assessment tool. Ordinal responses were analyzed using ordinal logistic regression. Scores per domain are provided as the mean Likert-score and the confidence intervals. All quantitative data were analyzed using Stata IC, version 15. Thematic analysis occurred for the focus groups with items grouped by theme and subtheme. Each section had three independent coders per focus group. Themes and subthemes were reviewed for frequency of response, repetition, and relatedness. Thematic analysis was cross-referenced across focus groups. Direct participant quotes were used to highlight themes.

## 3. Results

**Figure 1:**
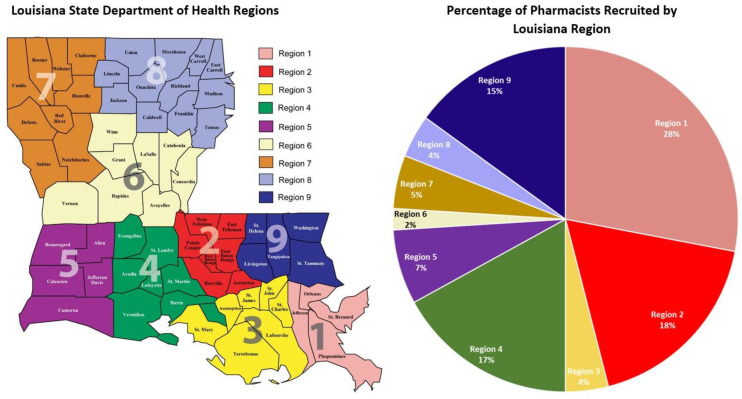
Community pharmacist recruitment per Louisiana Department of Health Region.

Fifty-four community pharmacists from across the state of Louisiana participated in the project, completing all study components. The percentages of pharmacist participants recruited per each Louisiana Department of Health Region may be found in Figure 1. While participants were representative of all nine Department of Health Regions, the majority of study participants practiced within Regions 1, 2, 4 and 9. Participants were an average age of 36 years old, were mostly female (69.5%), racially identified as Caucasian (57.4%), and had practiced pharmacy for an average of 11 years. Half of all participants practiced in a chain community pharmacy setting, while the remainder practiced in an independent community pharmacy, or other community pharmacy settings. Most participants reported receiving a COVID-19 vaccine. While only roughly 26% of the pharmacists reported being personally impacted by COVID-19 (testing positive, gaining or losing employment, participating in vaccine trials or other personal experiences), all but one participant reported having friends or family that were personally impacted by the virus. Participant baseline demographic information can be found in Table 1.

Mean score results in the bivariate analysis using the outcome of Role and Identity as well as Comfort Enforcing Vaccine Mandates are presented in Table 2. These outcomes were measured against participant demographic data, as well as regional Tertile-specific vaccination rates. Results regarding pharmacist identity and comfort enforcing vaccine mandates were generally positive. While no significant differences were seen, interesting trends were identified. In comparison to pharmacists employed at chain community pharmacies, those pharmacists employed at independent community pharmacies had more positive perceptions of their role and identity. However, pharmacists employed at independent community drug stores were less confident administering vaccines due to mandates than their pharmacist counterparts at chain pharmacies. Female participants and African American participants reported more comfort enforcing vaccine mandates. When comparing vaccination rate data per LDH Region Tertile to pharmacist confidence, pharmacists practicing in regions with the lowest Tertile of vaccine rates were more likely to be comfortable enforcing vaccine mandates but had the lowest perceived professional self-confidence. Yet, when looking at relative changes in vaccination rates from May 2021 to May 2022, pharmacists working in the LDH region with the highest relative change in vaccine uptake had the highest professional self-confidence but least comfort in enforcing vaccine mandates. Representing the data from May 2021, May 2022, and the relative change from May 2021 to May 2022 allows for reviews of temporal changes across region.

**Figure 2:**
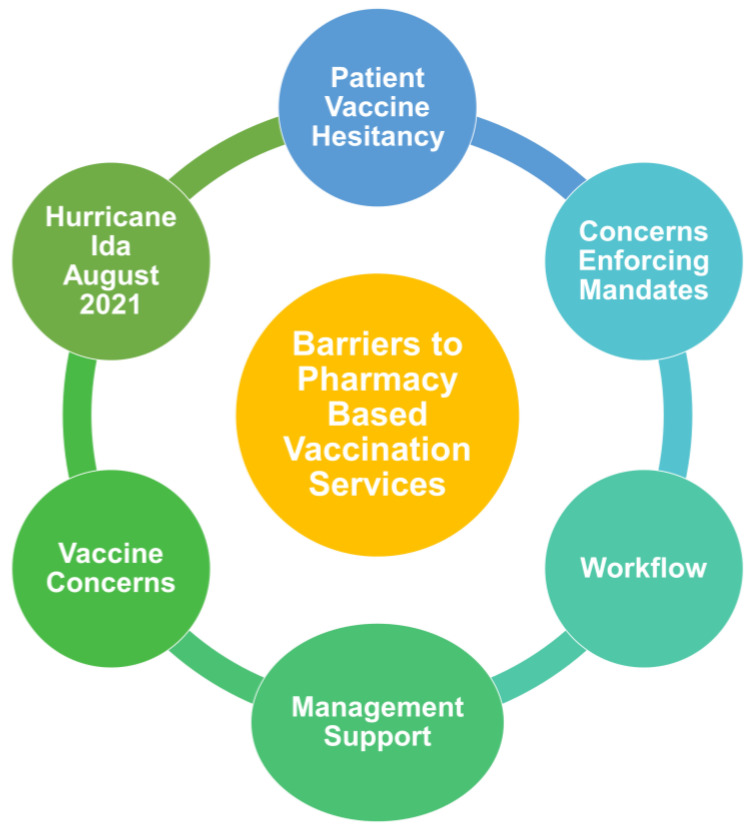
Barriers to pharmacy-based vaccination services: Major discussion themes captured through Louisiana community pharmacist focus groups.

During the COVID-19 based focus group discussions, several themes regarding barriers to vaccination were present. The resultant major themes are represented in Figure 2. One major discussion theme was pharmacy workflow. Pharmacists expressed concern regarding difficulty structuring their workflow to accommodate COVID-19 vaccinations, time constraints, vaccination scheduling, documentation, and proper storage of vaccines, including specialized refrigeration equipment. Many pharmacists expressed stress and exhaustion, citing understaffed pharmacies in the face of overwhelming workloads. One participant expressed, *“working in community pharmacy you know you’re doing more than just COVID shots, you’re doing flu shots, expanding shots, pick up, drive through COVID testing as well, and you know when it’s just you and one other person, it can be overwhelming.” (Focus group #1).* Some pharmacists reported that their management was inexperienced and unsupportive during the initial vaccine rollout. Several participants also expressed that they would like to be paid more for their increased workload, with some also citing difficulty obtaining insurance reimbursement for vaccinations. Participants working at independent pharmacies expressed more flexibility in terms of workflow and staffing decisions.

The majority of pharmacist participants (87%) reported encountering vaccine hesitancy from patients, with 73% reporting that patients sought counseling from the pharmacist before deciding to receive a COVID-19 vaccine. When asked if their patients’ concerns were similar or different from those surrounding other vaccines, 87% of pharmacists reported that COVID-19 vaccine concerns were different. Reported patient concerns included fear of side effects, beliefs that the vaccines were produced too quickly without enough research, healthcare system and government distrust, and misinformation sourced from social media and others. The participants cited a great need for patient education, with one pharmacist commenting, *“They really are believing all of the myths you hear, oh you’re altering my DNA, or you know, all of these untrue things about the vaccine, so really taking the time to sit down with the patient and explain to them, you know what the true benefits of getting the vaccine are…” (Focus group #2)*. Many participants described strong emotional responses from patients being vaccinated. One participant commented, *“I mean literally we had patients cry when we gave them vaccine because they were so happy and literally, the other day we gave a vaccine, and someone cried because they were so scared.” (Focus group #2)*

Pharmacists shared patient concerns with the rapid timing of COVID-19 vaccine development. Participants also remarked that consistent communication regarding COVID-19 and the vaccines was sometimes difficult to obtain, leaving pharmacists with unanswered questions while struggling to stay up to date with rapidly changing information. Pharmacists expressed difficulties concerning COVID-19 vaccine availability and stability. Participants reported their attempts to minimize wasting of vaccine doses, and described feelings of guilt when wasting of doses was inevitable. Community pharmacists noticed a shift in their practice as they vaccinated partially unwilling patients due to employer, school, or social event mandates. Some pharmacists expressed discomfort when vaccinating these patients, with one commenting, *“I’m not used to having this much upset over something I’ve done, you know—to you usually they’re thankful for the care they’re given.” (Focus group #1)*, and another remarking, *“Those are sometimes the hardest because they’re very unpleasant to us because this wasn’t their choice it’s you can get this vaccine and keep your job, or you can not get it and not have a job.” (Focus group #4)*. Finally, participants shared their experiences surrounding 2021’s Hurricane Ida. Several pharmacists were personally impacted by the natural disaster. Many reported losing vaccine stock due to extended losses of power and vaccine refrigeration capability. This, combined with additional delays in vaccine shipments, adversely impacted patient vaccination schedules. Some pharmacists reported losing several of their original patients to follow up while others reported gaining new patients from the storm-affected areas of the state.

## 4. Discussion

This study explored Louisiana community pharmacists’ confidence in their professional roles and identities, as well as their comfort in enforcing vaccine mandates. Results indicate that independent community pharmacists had a stronger sense of professional identity, as compared to their chain pharmacy counterparts, but were less comfortable enforcing vaccine mandates. Perhaps it is this particular pharmacy setting which truly promotes independence. Many independent pharmacies located within rural areas of the state may be one of few healthcare providers in their area [23]. During focus group discussions, pharmacists practicing at independent community stores described more flexibility in workplace decision making. While pharmacists working for a large chain corporation may become accustomed to centralized decision-making and following guidance from the top down, pharmacists at independent community stores exhibited a greater sense of autonomy, which they may extend to their patients in an empathetic way. Therefore, these pharmacists might reasonably feel less comfortable enforcing vaccine mandates on resistant or somewhat unwilling patients.

Vaccine mandates can be controversial as they pit individual personal autonomy against the health of the community. While this study’s pharmacist participants generally expressed comfort in administering vaccines due to mandates, global studies reviewing healthcare professionals’ attitudes regarding vaccination mandates reflect differing views. Healthcare workers in some nations supported mandates as an effective strategy to increase vaccination rates [42,43]. Others considered vaccine mandates to be a coercive action that does not address patient concerns, may not be an effective tool to increase vaccinations, and should be used as a last resort [43,44,45]. This is a view echoed by the World Health Organization (WHO), which warns against mandatory vaccinations unless all other options have been exhausted [46]. A multinational review suggests the COVID-19 pandemic as a catalyst for evolution of pharmacist professional role and identity [47]. As pharmacists navigate the crisis of the pandemic, they must create and adapt their roles to meet the needs of society. The roles pharmacists perform may influence their professional identity. As there is increased visibility of pharmacist’s essential public health contributions, both citizens and pharmacists alike may embrace the new roles for pharmacists. These functions may also become a part of pharmacist’s self-perceived identities [47].

Regional vaccination rate data were collected during the month of May in both 2021 and 2022. The date of May 2021 was chosen to provide a snapshot view of vaccine uptake across Louisiana regions once the COVID-19 phased vaccination rollout was completed, and the vaccines were available to the entire public. The collection date of May 2022 was selected to coincide with the end of the pharmacist educational intervention, which coincidentally occurred exactly one year later. The regional environment of the vaccine provider impacted self-perceived confidence and comfort in practice and implementation. For example, providers in the most vaccine-resistant parts of the state, defined as the lowest 3rd vaccination rates over the 12 months of the study, expressed the lowest level of self-perceived professional role but were the most comfortable in enforcing vaccine mandates in the beginning of the unrestricted post-phased rollout of the COVID-19 vaccine in May 2021. This trend continued until May 2022. Pharmacists practicing in regions with the greatest relative gains in vaccination continued to express more comfort in enforcing mandates but have relatively a lower perceived professional role. The general political and social distinctions in the regions across Louisiana impacted pharmacist professional perceptions and willingness to participate in government mandates throughout the first 18 months of the COVID-19 vaccine release.

There were several minority pharmacist participants in this study. While most participants self-identified as Caucasian, the remainder identified as African American, or Asian/Other. Sources indicate that African Americans make up less than 10% of the American pharmacist workforce [48]. It has been found that a diverse healthcare workforce which reflects the communities served can have positive effects on patient experience while reducing health disparities [49]. Considering COVID-19 and the disproportionate adverse outcomes experienced by African Americans, as well as the increased amount of vaccine hesitancy within this group, it was a vital benefit to include perspectives from a significant section of this demographic [50,51].

This study also explored the specific barriers that Louisiana pharmacists have faced during the COVID-19 pandemic. Focus group data revealed a concerning number of obstacles faced by community pharmacists. As in previous studies, participants voiced struggles with overwhelming workloads, low support from management, and combating patient vaccine hesitancy [6,7,8,9,28]. Most participants reported encountering vaccine hesitancy from patients and that patient concerns were different than those they have seen with other vaccines. While the reasons for vaccine hesitancy are multifaceted, specific conditions surrounding the COVID-19 pandemic gave rise to increased hesitancy. Similar to previous findings, patient concerns about side effects, rapid vaccine development, politicization of the pandemic, government mistrust as well as misinformation spread through forms of media were causes of vaccine hesitancy in Louisiana pharmacies [28,29,30,31,32,33]. Pharmacists were also challenged to resolve their own doubts and concerns regarding COVID-19 vaccines, as information changed rapidly. Despite these barriers, pharmacist participants expressed relatively strong professional identities, and remained committed to serving the public. This commitment was evidenced by the pharmacy workforce response to a natural disaster.

Because Louisiana is a coastal southern U.S. state, it is prone to natural disasters such as hurricanes, and in more recent years, tornadoes. Frequent hurricanes disrupt health services and exacerbate underlying disparities in the Southern region [52]. This was clearly seen in south Louisiana during and following Hurricane Ida of 2021. Of Louisiana’s nine regions, regions one and three were affected most by the hurricane, with some areas being without electricity for over two months. Participants noted that Ida and its aftermath impacted all aspects of pharmacy workflow from total electricity loss, to not receiving COVID-19 vaccine deliveries, to evacuations affecting both employees and patients. However, pharmacists remain one of the most accessible healthcare providers to communities and have demonstrated resourceful flexibility to meet the needs of patients during times of natural disaster, as well as during the COVID-19 pandemic [47].

### Limitations

This study was not without limitations. The study population sample was skewed towards a higher percentage of women than men. However, estimates of American pharmacist demographic data indicate that the field of pharmacy is trending towards a growing female pharmacist majority [48]. Additionally, our results also may not reflect the experiences or vaccination patterns of the roughly 6000 pharmacist practitioners within the entire state of Louisiana [35]. Many of this study’s participants were sourced from the greater New Orleans region, which has proven to be the most COVID-19 vaccine receptive community in Louisiana. Orleans Parish leads the state in per parish vaccination rates [53]. Nevertheless, the state of Louisiana is one of four states with the lowest percentage of fully vaccinated individuals, which indicates much room for pharmacist-driven improvement in vaccination rates [34]. Additionally, while geographically close, the four LDH regions from which most of the study’s pharmacists were recruited actually occupy three culturally distinct regions, providing a rich perspective across both rural and urban settings [36,37].

## 5. Conclusions

United States pharmacists have been providing vaccines to the community for more than 20 years. Although the decision to receive a vaccine is still a patient’s choice, as one of the most accessible healthcare professionals, pharmacists can be instrumental in providing patients with critical information to make informed choices when it comes to immunizations. Our study finds that Louisiana community pharmacists generally have high senses of professional identity and self-assurance. Although participant experiences and responses are influenced by the pandemic moment, pharmacists are performing under well-documented, challenging working conditions while providing pharmaceutical care to patients [6,7,8,9,10]. To be effective, pharmacists must have access to the resources needed to perform the duties asked of them, as well as receive appropriate compensation upon receiving additional responsibilities. Future initiatives in similar cohorts must recognize the barriers pharmacists are currently overcoming to provide care, and target solutions addressing those specific barriers to improve community pharmacist experiences and provision of pharmacy-based vaccination services. Despite facing both established and novel barriers during the COVID-19 pandemic, community pharmacists remain committed to their professional roles in educating communities, vaccinating patients and caring for vulnerable populations throughout the pandemic. Diverse pharmacist perspectives must be considered, and pharmacy-based vaccination services must be adequately supported to improve COVID-19 vaccine uptake.

## Figures and Tables

**Table 1:** Baseline Demographics (n = 54).

Variable	n (%)
Sex at birth (Male)	18 (30.5)
Race	
White	31 (57.4)
African-American	14 (25.9)
Asian/Other	9 (16.7)
Age (Mean/SD)	36 (10.6)
Age (Median/IQR)	31.5 (29–41)
Education (Highest Degree for Practice)	
BPharm	10 (18.5)
PharmD	44 (81.5)
Years as an RPH (Mean/SD)	11.11 (10.8)
Years as an RPH (Median/IQR)	7 (4–14)
Chain Community Pharmacy Practice Setting	27 (50)
Independent Community Pharmacy Practice Setting	20 (37)
Other Community Pharmacy Practice Setting	7 (13)
Received COVID-19 Vaccine	52 (96.3)
Employer Requires COVID-19 Vaccine	17 (31.5)
COVID-19 Impact	
Personally (+)	14 (25.9)
Friend or Family (+)	53 (98.1)

**Table 2:** Professional perceptions of pharmacists during the COVID-19 pandemic.

		Level of Role and Identity/Self Assurance	How Comfortable Are You to Enforce Vaccine Mandates
**What is your sex at birth?**	Male	4.09 (3.96, 4.22)	3.61 (3.02, 4.2)
	Female	4.09 (3.88, 4.29)	4.03 (3.67, 4.39)
**Race**	Caucasian	4.12 (3.89, 4.35)	3.6 (3.16, 4.04)
	African-American	4.08 (3.91, 4.25)	4.43 (3.89, 4.96)
	Asian or Other	3.99 (3.72, 4.25)	4.07 (3.47, 4.66)
**Highest Degree of Education Achieved for Pharmacy Practice**	BPharm	4.21 (4.03, 4.39)	3.9 (3, 4.8)
	PharmD and Above	4.06 (3.89, 4.23)	3.89 (3.56, 4.22)
**Does your employer require you to receive the COVID-19 vaccine?**	No	4.09 (3.89, 4.29)	3.81 (3.44, 4.18)
	Yes	4.08 (3.92, 4.24)	4.07 (3.51, 4.64)
**I have personally tested positive for COVID-19**	No or No Reply	4.05 (3.86, 4.24)	3.99 (3.61, 4.36)
	Yes	4.19 (4.07, 4.31)	3.63 (3.09, 4,17)
**Immunization Setting Primary**	Community Chain	3.97 (3.7, 4.23)	4.21 (3.85, 4.58)
	Community Independent	4.21 (4.1, 4.32)	3.55 (2.96, 4.14)
	Other Vaccine Venues and Events	4.21 (3.91, 4.52)	3.63 (2.61, 4.65)
**Practice Setting Primary**	Community Chain	3.94 (3.65, 4.22)	4.23 (3.85, 4.61)
	Community Independent	4.21 (4.11, 4.32)	3.48 (2.9, 4.06)
	Other Vaccine Venues and Events	4.23 (3.98, 4.49)	3.93 (3.14, 4.71)
**LDH Region Tertile Vacc Rates for May 2021**	T1: 23% to 27%	3.96 (3.58, 4.34)	4.08 (3.54, 4.63)
	T2: 29% to 35%	4.17 (4.05, 4.29)	3.85 (3.35, 4.35)
	T3: >42%	4.14 (3.98, 4.3)	3.71 (3.05, 4.37)
**LDH Region Tertile Vacc Rates for May 2022**	T1: 40% to 48%	3.96 (3.58, 4.34)	4.08 (3.54, 4.63)
	T2: 50% to 56%	4.17 (4.05, 4.29)	3.85 (3.35, 4.35)
	T3: >66%	4.14 (3.98, 4.3)	3.71 (3.05, 4.37)
**LDH Region Tertile Vacc Rates for May 2021 to May 2022 Relative**	T1: <59% (Lowest Change)	4.14 (3.98, 4.3)	3.71 (3.05, 4.37)
	T2: 63% to 67%	4.13 (4.01, 4.26)	3.76 (3.29, 4.22)
	T3: 72% to 80% (Highest Change)	3.98 (3.52, 4.43)	4.26 (3.7, 4.82)
5-point Likert scale, 1-Low level of assurance or confidence, 5-Highest level of assurance or confidence			

## Data Availability

Not applicable.
